# Identity and Specificity of *Rhizoctonia*-Like Fungi from Different Populations of *Liparis japonica* (Orchidaceae) in Northeast China

**DOI:** 10.1371/journal.pone.0105573

**Published:** 2014-08-20

**Authors:** Rui Ding, Xu-Hui Chen, Li-Jun Zhang, Xiao-Dan Yu, Bo Qu, Ru Duan, Yu-Feng Xu

**Affiliations:** College of Bioscience and Biotechnology, Shenyang Agricultural University, Shenyang, Liaoning, People’s Republic of China; The National Orchid Conservation Center of China; The Orchid Conservation & Research Center of Shenzhen, China

## Abstract

Mycorrhizal association is known to be important to orchid species, and a complete understanding of the fungi that form mycorrhizas is required for orchid ecology and conservation. *Liparis japonica* (Orchidaceae) is a widespread terrestrial photosynthetic orchid in Northeast China. Previously, we found the genetic diversity of this species has been reduced recent years due to habitat destruction and fragmentation, but little was known about the relationship between this orchid species and the mycorrhizal fungi. The *Rhizoctonia*-like fungi are the commonly accepted mycorrhizal fungi associated with orchids. In this study, the distribution, diversity and specificity of culturable *Rhizoctonia*-like fungi associated with *L. japonica* species were investigated from seven populations in Northeast China. Among the 201 endophytic fungal isolates obtained, 86 *Rhizoctonia*-like fungi were identified based on morphological characters and molecular methods, and the ITS sequences and phylogenetic analysis revealed that all these *Rhizoctonia*-like fungi fell in the same main clade and were closely related to those of *Tulasnella calospora* species group. These findings indicated the high mycorrhizal specificity existed in *L. japonica* species regardless of habitats at least in Northeast China. Our results also supported the wide distribution of this fungal partner, and implied that the decline of *L. japonica* in Northeast China did not result from high mycorrhizal specificity. Using culture-dependent technology, these mycorrhizal fungal isolates might be important sources for the further utilizing in orchids conservation.

## Introduction

The mycorrhizal association is ubiquitous but very important symbiosis in nature, which plays an essential role in the maintenance of most terrestrial ecosystems [1]. Over 90% of all plant species can form mycorrhizas with different kinds of fungi, and the existence of mycorrhizal fungi can confer to their hosts many adaptive advantages via improved water and nutrient/minerals uptake from the soil [2], [3], [4], enhanced plant growth [5], [6], reduced toxic element accumulation [1], [7], and increased resistence to pathogen damage [8]. Nowadays, based on their essential role in plant species and the whole ecosystems, more attention has been given to the potential of exploiting fungi for effective use [9], [10]. Although a large number of mycorrhizal fungi existed in plants, only a fraction has been described and explored to date, and their function in ecological systems remains indistinct.

The Orchidaceae, which is one of the largest and most diverse plant families, is distributed worldwide [11]. However, many orchid species have suffered dramatic declines in distribution and some species have become rare and endangered in recent decades [12]. Mycorrhizal association is known to be important to orchids because they depend on the presence of suitable fungal partners for seed germination and seedling development [13], [14]. Therefore, a complete understanding of the mycorrhizal fungi of the many threatened orchid species is required for conservation action plans [15].

Although molecular taxonomic identification of the endophytic fungi of orchid species has now revealed that the diversity of orchid associates is much complex [16], the study of the earliest-diverging orchid lineages and distribution of mycorrhizal fungal associates across orchid phylogeny supported that the ancestral state is an association to the *Rhizoctonia*-like fungi lineages [17], and orchid mycorrhizas are predominantly represented by associations between photosynthetic plants and *Rhizoctonia*-like fungi [15]. The *Rhizoctonia*-like fungi includes members of the Ceratobasidiaceae, Sebacinales and Tulasnellaceae [15], [17]. However, the taxonomy, diversity and distribution patterns of mycorrhizal fungi may vary considerably among different orchid species or different populations within the same species, ranging from very narrow specificity to little specificity [18]–[26]. Therefore, knowing the identity of the fungi that form mycorrhizas with orchids and the specificity of the relationships are of crucial importance for orchid ecology and conservation [27].

*Liparis japonica*, which is a terrestrial photosynthetic orchid species, has a wide geographic distribution in the tropical and subtropical areas such as China, Korea, Japan and Russia [28]. Historical record indicated that this species had a relatively wide distribution in Northeast China. Unfortunately, the number of *L. japonica* populations has declined sharply during the last several decades and many populations recorded previously has disappeared [29]. In a previous study, we assessed the genetic diversity and population genetic structure of *L. japonica* in Northeast China, and found potential restricted gene flow existed among populations due to habitat destruction and fragmentation [29]. To conserve surviving wild populations and reintroduce plants into declining populations requires a more complete understanding on the mycorrhizal fungal partners of *L. japonica* species.

In this study, we investigated in detail the taxonomy and diversity of culturable mycorrhizal fungi associated with *L. japonica* in Northeast China using morphological and ITS phylogenetic analysis. Seven different populations of *L. japonica* species were examined in order to evaluate how fungi diversity varied within and between habitats. Using a culture-dependent method, the fungal isolates obtained will be important sources for deep study and further utilizing in orchid conservation.

## Materials and Methods

### Study species and sampling

**Figure 1:**
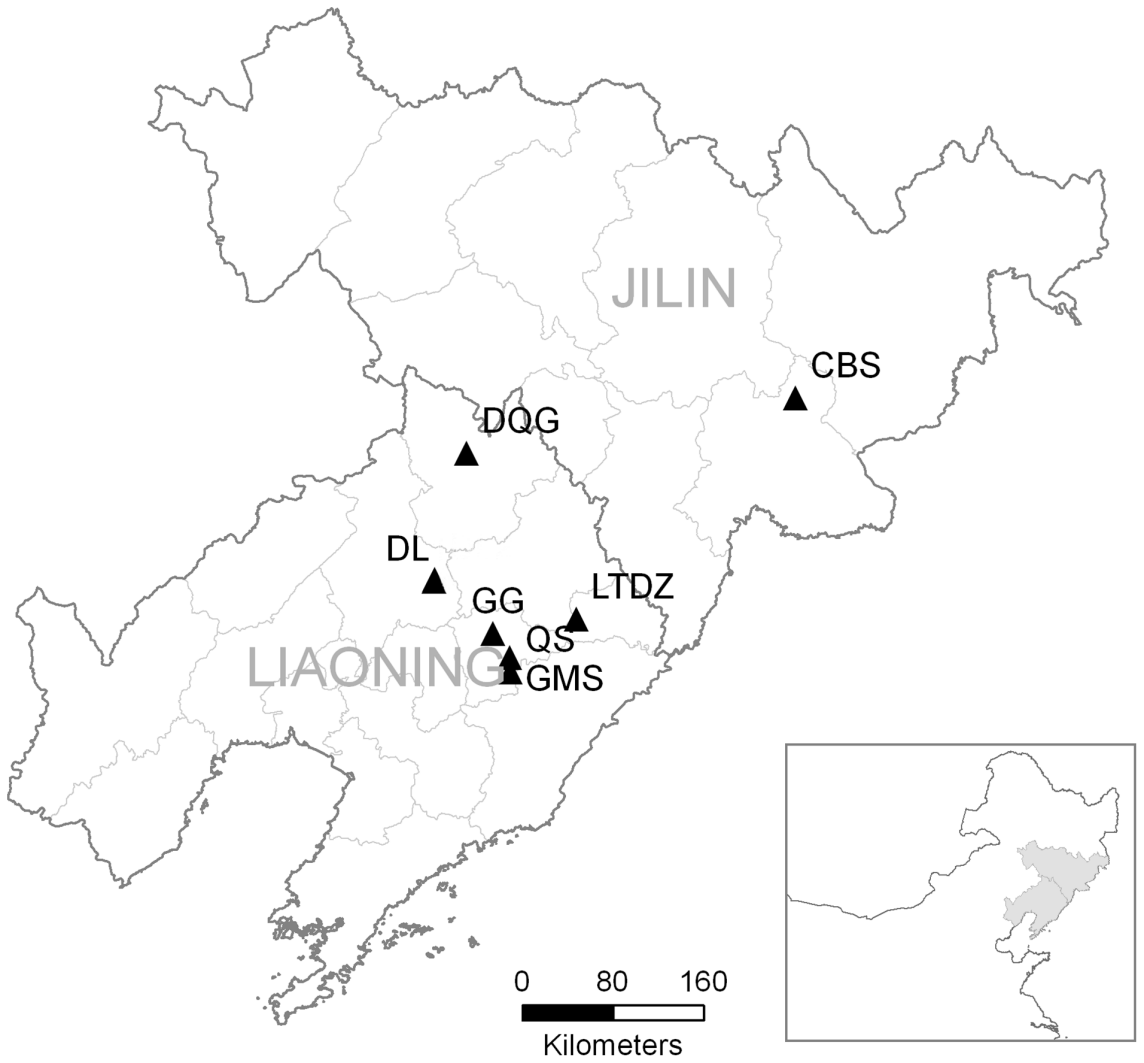
Locations of sampling sites of *Liparis japonica* in Northeast China.

185 individuals of *L. japonica* belonging to seven natural populations were collected in Northeast China at the flowering stage in June 2010 and July 2011, and the distances between these populations varied from 25 to 460 km. Details of locations and number of sampled individuals of each population were listed in Table 1 and Fig. 1. Plants were excavated with a clod of their surrounding soil and transferred to the laboratory. The field studies did not involve endangered or protected species, and no specific permissions were required for these locations.

**Table 1:** Location of the seven populations of *Liparis japonica* and the corresponding fungal isolates.

Population Code	Location	Longitude (E)	Latitude (N)	Altitude (m)	Samplesize	No. offungal isolates
DL	Dongling park, Shenyang,Liaoning	123°34′39″	41°49′53″	81	51	25
GG	Gaoguan, Benxi,Liaoning	124°02′32″	41°20′19″	274	30	30
GMS	Guanmenshan forest park, Benxi,Liaoning	124°09′30″	41°07′41″	438	18	27
CBS	Changbaishan nature reserve,Jilin	127°47′55″	42°30′35″	741	10	18
LTDZ	Laotudingzi nature reserve,Benxi, Liaoning	124°53′56″	41°18′09″	817	24	21
DQG	Daqinggou nature reserve, Fuxin,Liaoning	122°12′03″	42°43′55″	194	26	37
QS	Qianshan nature reserve,Anshan, Liaoning	123°07′48″	41°01′21″	163	26	43
Total					185	201

### Fungal isolation and morphological identification

Potential mycorrhizal fungi were isolated from the orchid plants and identified from pure culture. *L. japonica*, unlike many other temperate, terrestrial orchids which have thick roots and often produce abundant pelotons, has few active pelotons suitable for isolation. Thus, endophytic fungi were isolated from single hyphal tips emerging from sterilized root portions as in the isolation method described by [30]. Three roots per plant were carefully cleaned from the soil under running water, surface-sterilized in 0.1% (*v*/*v*) Mercuric chloride and 75% (*v*/*v*) ethanol for 3 min and 5 s respectively, and subsequently washed three times in sterile distilled water. Root sections of 3–5 mm thickness were obtained by cutting and placed in a petri dish with potato dextrose agar (PDA). Petri dishes were incubated at 25°C in the dark and observed for fungi growing every 2 days for at least 3 weeks. The growing colonies were separated onto fresh media for purity and this process was repeated three times. Classification of the endophytic fungi was based on their growth rate and morphological characteristics, including colonial morphology, production of conidiogenous cells, conidial size and dimension on PDA medium [31], and similar isolates were grouped into one morphotype. The *Rhizoctonia*-like fungal endophytes were recognized by the following characteristics: hyphas hyaline with constricted branch points, 2.5–7 µm diam; submerged growth in PDA; ellipsoid, globose or irregular monilioid cells 10–20×10–20 µm; colony creamy white to pale tan or orange, rubbery or leathery in appearance and texture [32]–[34].

### DNA extraction, amplification and sequencing

Genomic DNA was extracted from each *Rhizoctonia*-like fungi isolate as described by [35]. ITS sequences of rDNA were amplified using the following primer pairs: ITS1-OF/ITS4-OF [36]. PCRs were performed in 50-µL volumes containing approximately 10 µg of fungal genomic DNA, 1×PCR buffer (Sangon Biotech Co. Ltd), 1.5 mM MgCl_2_, 200 µM each of dNTP (dATP, dCTP, dGTP, and dTTP), 100 nM of each primer and 2.5 unit of *Taq* DNA polymerase (Sangon Biotech Co. Ltd). PCRs were carried out with a BIO-RAD T100^tm^ Thermalcycler with an initial incubation at 95°C for 5 min, followed by 35 cycles with denaturation at 95°C for 30 s, annealing at 60°C for 1 min and extension at 72°C for 1 min, and a final extension reaction at 72°C for 10 min. The PCR products were purified using the TaKaRa Agarose Gel DNA Purification Kit (TaKaRa, Dalian, China) and sequenced using the BigDye terminator v3.1 on an ABI 3730xl DNA Analyzer (Applied Biosystems, Forster City, California, USA). New ITS sequences from this study have been deposited in GenBank (the accession numbers is from KF537635 to KF537658).

### Data analysis

ITS sequences were analyzed with BLAST [37] against the NCBI sequence database to find the closest sequence matches in the GenBank database (http://www.ncbi.nlm.nih.gov). Genus and species of the database match were accepted whenever identity between our sequence and that of the database was greater than 97% [38]. Sequences used to construct phylogenetic trees were from organisms considered to be closely related to the mycorrhizal fungus of *L. japonica* based on BLAST searches, and additional orchid mycorrhizal fungi sequences (mainly based on [19]) were also chosen for phylogenetic analysis. Selected sequences were aligned using the program Clustal_X 2.1 [39]. Alignments were checked for ambiguities and adjusted manually when necessary and the alignment gaps were treated as missing information.

Phylogenetic analyses were estimated using maximum parsimony (MP), Neighbor-joining (NJ) and Bayesian inference (BI) methods in PAUP* version 4.0b10 [40], MEGA version 4 [41] and MrBayes version 3.0b4 [42], respectively. For MP analysis, the bootstrap support values were estimated by 1000 replicates with 10 random sequence additions and tree-bisection-reconnection (TBR) branch swapping. For NJ analysis, genetic distances were calculated using the Kimura two-parameter model, and the bootstrap support values were estimated by 1000 replicates. Prior to the Bayesian analysis, the Akaike information criterion (AIC) in Modeltest version 3.7 [43] was used to select the best-fit model of molecular evolution for each dataset. For BI analysis, four chains of the Markov Chain Monte Carlo (MCMC) chains were run, sampling one tree every 1000 generations for 3,000,000 generations, starting with a random tree. Bayesian posterior probabilities (PP) were calculated from the majority rule consensus of the tree. The sequence of *Septobasidium carestianum* (GenBank accession number DQ241448) was selected as outgroup [21].

## Results

### Fungal distribution and morphological diversity

**Figure 2:**
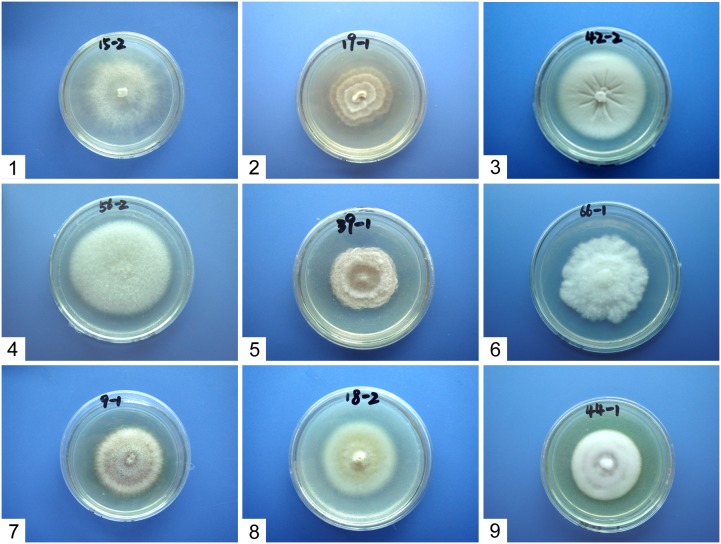
The nine morphotypes of endophytes isolated from *Liparis japonica* at seven populations. (morphotype I: *Rhizoctonia* sp., morphotype II: *Phomopsis* sp., morphotype III: *Verticillium* sp., morphotype IV: *Fusarium* sp., morphotype V: *Chaetomium* sp., morphotype VI: *Gliocladium* sp., morphotype VII: *Cylindrocarpon* sp., morphotype VIII: *Phialophora* sp., morphotype IX: *Paecilomyces* sp.).

After isolation and purification, a total of 201 isolates of endophytic fungi from 185 plants were obtained from seven *L. japonica* populations. According to their morphological characters and growth rate on PDA medium, fungal isolates were classified into nine morphotypes with four to seven morphotypes in each population (Table 2). The isolates in morphotypes I and II were observed in all populations studied and represented the majority of the fungal community (61.7%), while isolates from other morphotypes were not common and occurred only at one or a few populations (Table 2). Morphological characters and detailed descriptions of the nine morphotypes were given in Fig. 2 and Table 3, and according to the colony and micro-morphological characters, we identified morphotype I as *Rhizoctonia* sp., morphotype II as *Phomopsis* sp., and morphotype III to IX as *Verticillium* sp., *Fusarium* sp., *Chaetomium* sp., *Gliocladium* sp., *Cylindrocarpon* sp., *Phialophora* sp., and *Paecilomyces* sp., respectively. Only the *Rhizoctonia*-like isolates in morphotype I were given codes and subjected to further collection of mycelium for DNA extraction and phylogenetic analysis.

**Table 2:** Distribution of 201 fungal isolates in different morphotypes.

Morphotype	No. of isolates in different morphotype							Total	Percentage (%)
	DL	GG	GMS	CBS	LTDZ	DQG	QS		
I	7	8	9	12	7	21	22	86	42.8
II	3	5	14	3	2	5	6	38	18.9
III	5	5	1	0	3	4	6	24	11.9
IV	6	3	1	0	5	3	3	21	10.4
V	2	5	2	2	3	0	4	18	9.0
VI	1	2	0	0	1	2	1	7	3.5
VII	0	2	0	0	0	0	1	3	1.5
VIII	0	0	0	1	0	2	0	3	1.5
IX	1	0	0	0	0	0	0	1	0.5
Total	25	30	27	18	21	37	43	201	

**Table 3:** Morphological characters of the 9 morphotypes of fungal isolates from *Liparis japonica.*

Morphotype	Colony color	Colonytexture	Growth rate(mm day^−1^)	Aerial mycelium	Conidialshape	ConidiaSize (µm)
I	off-white	leathery, compact	11.9±1.5	absent	–	–
II	pink to tan	leathery,radialdraped	4.9±0.5	absent	elliptic cylindrical	6–8×3–4
III	white or buff	cottony,slightly loose	5.7±0.4	scarce	fusiform	5×1–1.5
IV	white	lanose,loose	11.8±0.5	abundant	ovate,cylindrical	cylindrical:15–20×5–10ovale: 4×3
V	white atfirst,then olive-green	pulvinate, compact	4.8±0.5	absent	limoniform	8–9×4–5
VI	white	cottony,slightlyloose	5.0±0.2	abundant	elliptic	6–8×3–5
VII	pale tan	cottony, compact	5.2±0.2	abundant	clavate,elliptic	40×5–10
VIII	Pale taupe	felted,compact	3.4±0.4	scarce	ovate	3–5×2.5
IX	lilac	felted,compact	3.3±1.7	abundant	elliptic	4–5×2

Of the 185 individuals of *L. japonica* samples, each of 180 samples gave rise to one *Rhizoctonia*-like isolate, whereas the other five samples gave rise to two *Rhizoctonia*-like isolates, and no sample gave rise to three or more *Rhizoctonia*-like isolates.

### Phylogenetic analysis

About 640-bp portions of the ITS were sequenced for the 24 representative isolates of morphotype I, with 3 to 4 isolates within each population. All sequences identified by BLAST in NCBI were from Tulasnellaceae strains. Thus, the representative *Tulasnella* spp. that have been identified were selected for phylogenetic analysis with the sequences obtained in this study, and a total of 54 different sequences were used.

**Figure 3:**
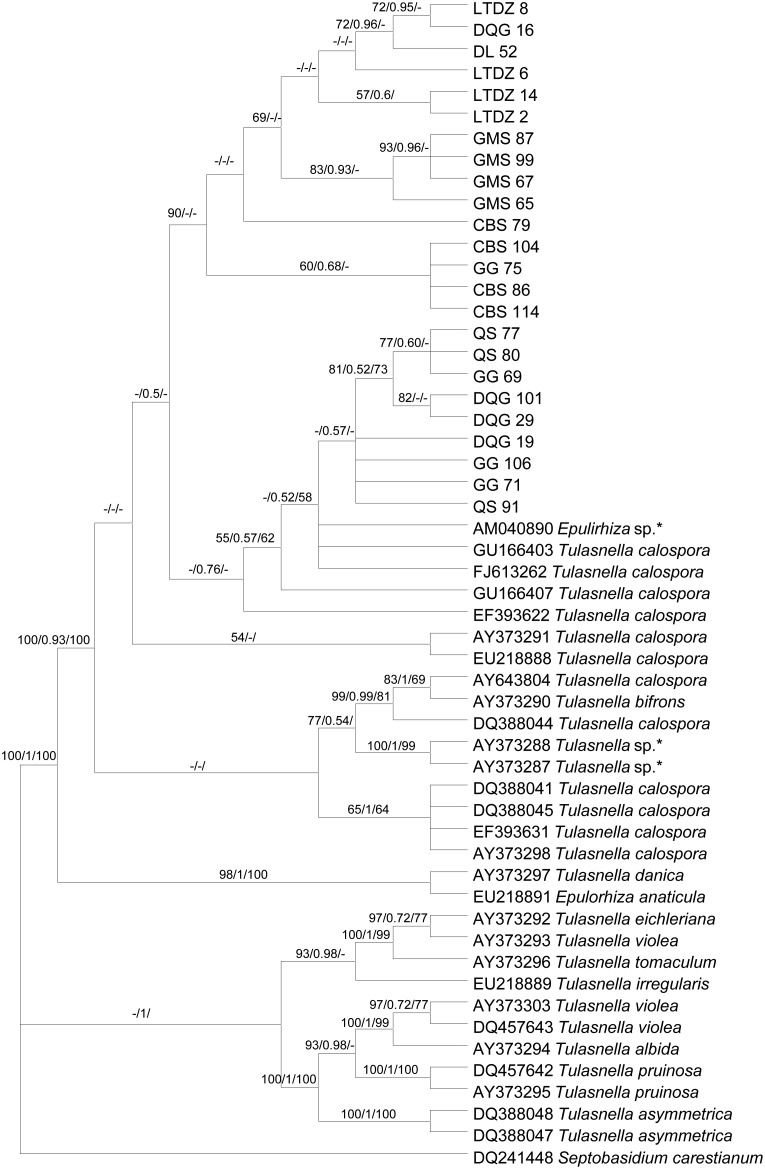
Strict consensus tree of Maximum parsimony (MP) based on ITS sequences of representative isolates of *Rhizoctonia*-like fungi isolated from *Liparis japonica* and identified *Tulasnella* spp. and *Epulorhiza* spp. Numbers at the nodes are MP bootstrap percentages, Bayesian posterior probabilities and Neighbour-joining support percentages (≥50%), respectively. “-” indicates node not supported in Bayesian inference and Neighbour-joining analyses. An asterisk indicates fungi isolated from *Liparis lilifolia* and *L. loeselii* (other *Liparis* species). The *Septobasidium carestianum* sequence was designated as outgroup for rooting the trees. Sequences obtained from GenBank were shown with accession numbers.

The overall topology of the trees produced with Maximum parsimony (MP), Bayesian inference (BI) and Neighbour-joining (NJ) methods were similar based on our ITS data (Fig. 3). The MP strict consensus tree (Fig. 3) showed that fungal associates of *L. japonica* all belonged to the genus *Tulasnella*, and all isolates belonged to a single major clade. Some mycorrhizal isolates from other orchid species which have been identified as *T. calospora*, and a mycorrhizal isolate (*Epulorhiza* sp., AM040890; [44]) from *L. loeselii* species in Hungary also fell in this major clade.

## Discussion

Compared with the direct DNA amplification technique, identification of orchid mycorrhizal fungi with the pure culture technique has been criticized because of the bias toward fast-growing and easily cultured fungi [15]. However, the culture-dependent technique is still used in a vast majority of orchid mycorrhizal fungi studies, mainly because fungi partners of these plants were usually easy to isolate and no significant differences were found between the pure culture technique and direct DNA amplification technique [20], [23], [45]–[47]. Unlike many other temperate, terrestrial orchids which have thick roots and often produce abundant pelotons, *L. japonica* has few active pelotons suitable for isolation and direct PCR identification, so we used standard culture method for fungi identification in this study, and the results were credible by the striking observation of the same fungal clade isolated across such a wide geographical distribution of *L. japonica*. Moreover, although the molecular method is powerful for fungi identification, isolation and cultivation of the mycorrhizal fungi isolates are still needed for deep study and further utilizing in orchids conservation.

In the present study, various endophytic fungi were isolated from roots of *L. japonica* and identified by morphological and the rDNA ITS sequences, and most of the identified endophytes of *L. japonica* are Basidiomycetes and Ascomycetes. In general, endophytic fungi in orchid plants are very abundant, but it seems likely that only *Rhizoctonia*-like fungi species may form mycorrhizal associations with host [48]. In this study, 86 endophyte fungi isolates belonging to the *Tulasnella* genus were isolated (42.8%) based on morphological characters and phylogenetic relationships reconstructed from ITS sequence, and they existed in every *L. japonica* population. Although some endophytes have been shown to be able to increase the tolerance of some plant species [49], little is known about the role of endophytes for orchid performance, and the non-*Rhizoctonia*-like endophytic fungi also deserve additional study.

The specificity of orchid mycorrhizal associations has important implications for orchid biology, conservation and restoration of orchid populations [50]. Specific fungal partners may lead to enhanced seed germination rates, and the efficiency of nutrient exchange between partners may be heightened with specific plant-fungus combinations [45]. Our results showed that all mycorrhizal fungi isolates obtained belonged to a single clade of *Tulasnella*, and the molecular analysis showed that they are all representatives of the same species, *T. calospora*, which indicates that the relationship between *L. japonica* and its mycorrhizal fungus is very specific regardless of habitats, at least in Northeast China. There are more and more reports showed that the degree of specificity can vary among species independent on orchid types [19], [22]. There is also evidence that a preference exists among some orchids for specific fungal partners, and some photosynthetic orchids, even when sampled over a wide range, have a single dominant mycorrhizal fungus, for example, *Spiranthes sinensis* [33], *Goodyera pubescens* [51], and *Pterostylis nutans* [52]. Our results also supported that high specificity in widespread and photosynthetic orchids may be more common than previously thought. Moreover, the phenomenon that multiple fungal taxa existed in a single root system was detected in some photosynthetic orchids which indicated that multiple fungi in a single root system may occur more often in photosynthetic than in mycoheterotrophic orchids [26]. While our results showed that this characteristic is not universal in orchid mycorrhizal systems.

From another perspective, the mycorrhizal specificity we observed might not be accurate for the reason that our results were based on a single collection of root tissue in adult *L. japonica* plants. However, the diversity and identify of orchid mycorrhizal fungi may vary according to different life stages of plant. Specificity during germination and early development has been detected in orchids, whereas the adult stage of plant growth in the same species can have less specific fungal partners [13], [51], [53]. Accordingly, fungi required for germination and recruitment of *L. japonica* might be different from those associated with adult plants, and this will be the next logical steps to investigate the pattern of mycorrhizal association.

Although *L. japonica* had a strong preference for *T. calospora* species across the sampled range, the fungus did not have an equivalent specificity. *T. calospora* has been shown to be a very common mycorrhizal fungus of many terrestrial orchid species [15], and the fact that *T. calospora* stains were present in different populations of *L. japonica* in Northeast China in our study and many other orchids in other regions of the world indicates its wide distribution in different ecological environments. Because of this, we considered that the decline of *L. japonica* in Northeast China was not resulted from fungal specificity, although there exists a point that a dependence on narrowly specific interactions with fungi and pollinator may predispose many orchids to become rare [15], [45], [54].

## References

[1] Smith SE, Read DJ (2008) Mycorrhizal Symbiosis. New York: Academic Press. 19–41.

[2] Gardes M (2002) An orchid-fungus marriage: physical promiscuity, conflict and cheating. New Phytol 154: 4–7.

[3] Allen MF (2007) Mycorrhizal fungi: highways for water and nutrients in arid soils. Vadose Zone J 6: 291–297.

[4] Leigh J, Hodge A, Fitter AH (2009) Arbuscular mycorrhizal fungi can transfer substantial amounts of nitrogen to their host plant from organic material. New Phytol 181: 199–207. 18811615 10.1111/j.1469-8137.2008.02630.x

[5] Artursson V, Finlay RD, Jansson JK (2006) Interactions between arbuscular mycorrhizal fungi and bacteria and their potential for stimulating plant growth. Environ Microbiol 8: 1–10. 16343316 10.1111/j.1462-2920.2005.00942.x

[6] Porras-Soriano A, Soriano-Martίn ML, Porras-Piedra A, Azcόn R (2009) Abruscular mycorrhizal fungi increased growth, nutrient uptake and tolerance to salinity in olive trees under nursery conditions. J Plant Physiol 166: 1350–1359. 19342122 10.1016/j.jplph.2009.02.010

[7] Fomina MA, Alexander IJ, Colpaert JV, Gadd GM (2005) Solubilization of toxic metal minerals and metal tolerance of mycorrhizal fungi. Soil Biol Biochem 37: 851–866.

[8] Yao MK, Tweddell RJ, Désilets H (2002) Effect of two vesicular-arbuscular mycorrhizal fungi on the growth of micropropagated potato plantlets and on the extent of disease caused by *Rhizoctonia solani* . Mycorrhiza 12: 235–242. 12375134 10.1007/s00572-002-0176-7

[9] Harms H, Schlosser D, Wick LY (2011) Untapped potential: exploiting fungi in bioremediation of hazardous chemicals. Nat Rev Microbiol 9: 177–192. 21297669 10.1038/nrmicro2519

[10] Zeng Y, Guo LP, Chen BD, Hao ZP, Wang JY, et al (2013) Arbuscular mycorrhizal symbiosis and active ingredients of medicinal plants: current research status and prospectives. Mycorrhiza 23: 253–265. 23417725 10.1007/s00572-013-0484-0

[11] Dressler RL (1993) Phylogeny and classification of the orchid family. Portland: Dioscorides Press.

[12] Swarts ND, Dixon KW (2009) Perspectives on orchid conservation in botanic gardens. Trends Plant Sci 14: 590–598. 19733499 10.1016/j.tplants.2009.07.008

[13] Bidartondo MI, Read DJ (2008) Fungal specificity bottlenecks during orchid germination and development. Mol Ecol 17: 3707–3716. 18627452 10.1111/j.1365-294X.2008.03848.x

[14] Rasmussen HN (2002) Recent developments in the study of orchid mycorrhiza. Plant Soil 244: 149–163.

[15] Dearnaley JDW, Martos F, Selosse M-A (2012) Orchid mycorrhizas: molecular ecology, physiology, evolution and conservation aspects. In: Hock B, editors. Fungal Association, 2nd edn. Berlin: Springer-Verlag. 207–230.

[16] Tondello A, Vendramin E, Villani M, Baldan B, Squartini A (2012) Fungi associated with the southern Eurasian orchid *Spiranthes spiralis* (L.) Chevall. Fungal Biol 116: 543–549. 22483052 10.1016/j.funbio.2012.02.004

[17] Yukawa T, Ogura-Tsujita Y, Shefferson RP, Yokoyama J (2009) Mycorrhizal diversity in *Apostasia* (Orchidaceae) indicates the origin and evolution of orchid mycorrhiza. Am J Bot 96: 1997–2009. 21622320 10.3732/ajb.0900101

[18] Chen J, Zhang LC, Xing YM, Wang YQ, Xing XK, et al (2013) Diversity and taxonomy of endophytic Xylariaceous fungi from medicinal plants of *Dendrobium* (Orchidaceae). PLOS ONE 8: e58268. 23472167 10.1371/journal.pone.0058268 PMC3589337

[19] McCormick MK, Whigham DF, O’Neill J (2004) Mycorrhizal diversity in photosynthetic terrestrial orchids. New Phytol 163: 425–438. 10.1111/j.1469-8137.2004.01114.x 33873625

[20] Otero JT, Flanagan NS, Herre EA, Ackerman JD, Bayman P (2007) Widespread mycorrhizal specificity correlates to mycorrhizal function in the neotropical, epiphytic orchid *Ionopsis utricularioides* (Orchidaceae). Am J Bot 94: 1944–1950. 21636389 10.3732/ajb.94.12.1944

[21] Nontachaiyapoom S, Sasirat S, Manoch L (2010) Isolation and identification of *Rhizoctonia*-like fungi from roots of three orchid genera, *Paphiopedilum*, *Dendrobium*, and *Cymbidium*, collected in Chiang Rai and Chiang Mai provinces of Thailand. Mycorrhiza 20: 459–471. 20107843 10.1007/s00572-010-0297-3

[22] Jacquemyn H, Deja A, De hert K, Bailarote BC, Lievens B (2012) Variation in mycorrhizal associations with Tulasnelloid fungi among populations of five *Dactylorhiza* species. PLOS ONE 7: e42212. 22870305 10.1371/journal.pone.0042212 PMC3411701

[23] De LongJR, Swarts ND, Dixon KW, Egerton-Warburton LM (2013) Mycorrhizal preference promotes habitat invasion by a native Australian orchid: *Microtis media* . Ann Bot 111: 409–418. 23275632 10.1093/aob/mcs294 PMC3579446

[24] Xing X, Ma X, Deng Z, Chen J, Wu F, et al (2013) Specificity and preference of mycorrhizal associations in two species of the genus *Dendrobium* (Orchidaceae). Mycorrhiza 23: 317–324. 23271631 10.1007/s00572-012-0473-8

[25] Oliveira SF, Bocayuva MF, Veloso TGR, Bazzolli DMS, da Silva CC, et al (2014) Endophytic and mycorrhizal fungi associated with roots of endangered native orchids from the Atlantic Forest, Brazil. Mycorrhiza 24: 55–64. 23812655 10.1007/s00572-013-0512-0

[26] Pandey M, Sharma J, Taylor DL, Yadon VL (2013) A narrowly endemic photosynthetic orchid is non-specific in its mycorrhizal associations. Mol Ecol 22: 2341–2354. 23432406 10.1111/mec.12249

[27] McCormick MK, Jacquemyn H (2014) What constrains the distribution of orchid populations? New Phytol 202: 392–400.

[28] Editorial Committee of the Flora of China (1992) Flora of China. Beijing: Science Press. 18: 62–64.

[29] Chen XH, Guan JJ, Ding R, Zhang Q, Ling XZ, et al (2013) Conservation genetics of the endangered terrestrial orchid *Liparis japonica* in Northeast China based on AFLP markers. Plant Syst Evol 299: 691–698.

[30] Curtis JT (1939) The relation of specificity of orchid mycorrhizal fungi to the problem of symbiosis. Am J Bot 26: 390–399.

[31] Wei YK, Gao YB, Zhang X, Su D, Wang YH, et al (2007) Distribution and diversity of *Epichloë/Neotyphodium* fungal endophytes from different populations of *Achnatherum sibiricum* (Poaceae) in the Inner Mongolia Steppe, China. Fungal Divers 24: 329–345.

[32] Currah RS, Sherburne R (1992) Septal ultrastructure of some fungal endophytes from boreal orchid mycorrhizas. Mycol Res 96: 583–587.

[33] Masuhara G, Katsuya K (1994) *In situ* and *in vitro* specificity between *Rhizoctonia* spp. and *Spiranthes-sinensis* (Persoon) Ames var. *amoena* (M. Bieberstein) Hara (Orchidaceae). New Phytol 127: 711–718. 10.1111/j.1469-8137.1994.tb02974.x 33874389

[34] Ma M, Tan TK, Wong SM (2003) Identification and molecular phylogeny of *Epulorhiza* isolates from tropical orchids. Mycol Res 107: 1041–1049. 14563130 10.1017/s0953756203008281

[35] Cenis JL (1992) Repid extraction of fungal DNA for PCR amplification. Nucleic Acids Res 20: 2380. 1594460 10.1093/nar/20.9.2380 PMC312363

[36] Taylor DL, McCormick MK (2008) Internal transcribed spacer primers and sequences for improved characterization of basidiomycetous orchid mycorrhizas. New Phytol 177: 1020–1033. 18086221 10.1111/j.1469-8137.2007.02320.x

[37] Altschul SF, Madden TL, Schaffer AA, Zhang J, Zhang Z, et al (1997) Gapped BLAST and PSI-BLAST: A new generation of protein database search programs. Nucleic Acids Res 25: 3389–3402. 9254694 10.1093/nar/25.17.3389 PMC146917

[38] Sánchez MS, Bills GF, Zabalgogeazcoa I (2008) Diversity and structure of the fungal endophytic assemblages from two sympatric coastal grasses. Fungal Divers 33: 87–100.

[39] Thompson JD, Gibson TJ, Plewniak F, Jeanmougin F, Higgins DG (1997) The Clustal_X windows interface: flexible strategies for multiple sequence alignment aided by quality analysis tools. Nucleic Acids Res 25: 4876–4882. 9396791 10.1093/nar/25.24.4876 PMC147148

[40] Swofford DL (2002) PAUP*: phylogenetic analysis using parsimony (* and other methods), version 4.0b10. Sinauer, Sunderland.

[41] Tamura K, Dudley J, Nei M, Kumar S (2007) MEGA4: molecular evolutionary genetics analysis (MEGA) software version 4.0. Mol Biol Evol 24: 1596–1599. 17488738 10.1093/molbev/msm092

[42] Ronquist F, Huelsenbeck JP (2003) MrBayes 3: Bayesian phylotenetic inference under mixed models. Bioinformatics 19: 1572–1574. 12912839 10.1093/bioinformatics/btg180

[43] Posada D, Crandall KA (1998) Modeltest: testing the model of DNA substitution. Bioinformatics 14: 817–818. 9918953 10.1093/bioinformatics/14.9.817

[44] Illyés Z, Halász K, Rudnóy S, Ouanphanivanh N, Garay T, et al (2009) Changes in the diversity of the mycorrhizal fungi of orchids as a function of the water supply of the habitat. J Appl Bot Food Qual 83: 28–36.

[45] Bonnardeaux Y, Brundrett M, Batty A, Dixon K, Koch J, et al (2007) Diversity of mycorrhizal fungi of terrestrial orchids: compatibility webs, brief encounters, lasting relationships and alien invasions. Mycol Res 111: 51–61. 17289365 10.1016/j.mycres.2006.11.006

[46] Roche SA, Carter RJ, Peakall R, Smith LM, Whitehead MR, et al (2010) A narrow group of monophyletic *Tulasnella* (Tulasnellaceae) symbiont lineages are associated with multiple species of *Chiloglottis* (Orchideacea): implications for orchid diversity. Am J Bot 97: 1313–1327. 21616884 10.3732/ajb.1000049

[47] Kohout P, Těšitelová T, Roy M, Vohník M, Jersáková J (2013) A diversity fungal community associated with *Pseudorchis albida* (Orchidaceae) roots. Fungal Ecol 6: 50–64.

[48] Suárez JP, Weiβ M, Abele A, Garnica S, Oberwinkler F, et al (2006) Diverse tulasnelloid fungi form mycorrhizas with epiphytic orchids in an Andean cloud forest. Mycol Res 110: 1257–1270. 17081740 10.1016/j.mycres.2006.08.004

[49] Rodriguez RJ, Henson J, Volkenburgh EV, Hoy M, Wright L, et al (2008) Stress tolerance in plants via habitat-adapted symbiosis. ISME J 2: 404–416. 18256707 10.1038/ismej.2007.106

[50] Dearnaley JDW (2007) Further advances in orchid mycorrhizal research. Mycorrhiza 17: 475–486. 17582535 10.1007/s00572-007-0138-1

[51] McCormick MK, Whigham DF, Sloan D, O’Malley K, Hodkinson B (2006) Orchid-fungus fidelity: a marriage meant to last? Ecology 87: 903–911. 16676534 10.1890/0012-9658(2006)87[903:ofammt]2.0.co;2

[52] Irwin MJ, Bougoure JJ, Dearnaley JDW (2007) *Pterostylis nutans* (Orchidaceae) has a specific association with two *Ceratobasidium* root-associated fungi across its range in eastern Australia. Mycoscience 48: 231–239.

[53] Zellter LW, Piskin KA (2011) Mycorrhizal fungi from protocorms, seedlings and mature plants of the eastern prairie fringed orchid, *Platanthera leucophaea* (Nutt.) Lindley: A comprehensive list to augment conservation. Am Midl Nat 166: 29–39.

[54] Swarts NS, Sinclair EA, Francis A, Dixon KW (2010) Ecological specialization in mycorrhizal symbiosis leads to rarity in an endangered orchid. Mol Ecol 19: 3226–3242. 20618899 10.1111/j.1365-294X.2010.04736.x

